# Reference interval of parathyroid hormone based on the Korea National Health and Nutrition Examination Survey 2024

**DOI:** 10.1186/s12902-026-02292-0

**Published:** 2026-04-28

**Authors:** Rihwa Choi, Sung-Eun Cho, Sang Gon Lee

**Affiliations:** 1 Laboratory Medicine Center, GC Labs, Yongin, Gyeonggi 16924 Republic of Korea; 2 Department of Laboratory Medicine and Genetics, Sungkyunkwan University School of Medicine, Seoul, Republic of Korea; 3 Endocrine Substance Analysis Center (ESAC), GC Labs, Yongin, Gyeonggi Republic of Korea

**Keywords:** Bone health, Parathyroid hormone, Reference interval, National health survey

## Abstract

**Background:**

Parathyroid hormone (PTH) plays a pivotal role in calcium–phosphate metabolism and bone health; however, interpretation of PTH concentrations remains challenging because of biological variability and substantial analytical heterogeneity among assays. In Korea, population-based data on PTH reference intervals are limited, and assay-specific reference intervals have not been well established.

**Methods:**

We evaluated the reference interval of serum PTH using data from the Korea National Health and Nutrition Examination Survey (KNHANES) 2024. A stepwise, biologically informed selection process was applied to define reference populations by progressively excluding participants with conditions known to influence PTH metabolism, including impaired renal function, vitamin D insufficiency, and abnormalities in mineral-related biochemical parameters (calcium, magnesium, phosphate, and alkaline phosphatase).

**Results:**

Among the 6,997 participants in the KNHANES 2024 dataset, serum PTH measurements were available for 4,502 adults aged ≥ 40 years. The high prevalence of supplement use and age-restricted sampling limited the size of suitable reference populations. The number of survey-design–valid participants frequently fell below the recommended threshold for reference interval estimation (< 120) after applying criteria for vitamin D sufficiency, mineral homeostasis, and age- and sex-specific alkaline phosphatase. Among 155 participants without a history of disease, not using dietary supplements, not pregnant, and with an estimated glomerular filtration rate ≥ 60 mL/min/1.73 m², the unweighted and survey-weighted 2.5th–97.5th reference intervals of PTH were 21.8–65.0 pg/mL and 20.5–67.7 pg/mL, respectively, when vitamin D status and serum calcium, magnesium, and phosphate levels were not considered.

**Conclusions:**

Future population-based studies incorporating broader age ranges and comprehensive biochemical profiling are needed to refine population- and assay-specific PTH reference intervals and to clarify their implications for bone health.

**Clinical trial number:**

Not applicable.

**Supplementary Information:**

The online version contains supplementary material available at 10.1186/s12902-026-02292-0.

## Introduction

Parathyroid hormone (PTH) plays a central role in calcium–phosphate homeostasis and bone metabolism, and measurement of PTH is routinely used in the evaluation of parathyroid disorders, including primary and secondary hyperparathyroidism and hypoparathyroidism, as well as conditions such as chronic kidney disease–mineral and bone disorder and osteoporosis [1–5]. Despite its widespread clinical use, interpretation of serum PTH concentrations remains challenging because of substantial biological variability and marked analytical heterogeneity among available PTH assays [6–8]. Importantly, PTH reference intervals should be established in an assay-specific as well as ethnicity-specific manner [6–9]. Circulating PTH exists in multiple molecular forms, and differences in assay generation, antibody combinations, and targeted epitopes result in limited interchangeability of PTH measurements across analytical platforms [7–10]. Despite ongoing efforts by the IFCC Committee on Bone Metabolism since 2017 to standardize PTH measurement, full standardization has not yet been achieved, and the proposed mass spectrometry–based reference method remains under validation [8, 9, 11–14]. Consequently, reference intervals derived from one assay cannot be universally applied to others, underscoring the need for assay-specific interpretation of PTH results [15–18].

Accurate interpretation of PTH further requires reference populations defined under physiologically appropriate conditions. Renal function, vitamin D status, and mineral-related biochemical parameters—including serum calcium (Ca), magnesium (Mg), and phosphate (P)—are major determinants of PTH secretion [15, 18]. Consequently, reference intervals derived from unselected populations may not reflect a true physiological baseline and may result in misclassification [19–21]. Although several studies have evaluated PTH reference intervals in Western populations, data for the Korean population remain limited, particularly studies that incorporate both biological selection criteria and assay-specific considerations [15–18]. Ethnic differences in dietary patterns, vitamin D status, supplement use, and bone health characteristics further limit the applicability of reference intervals established in non-Korean populations [15–21].

The Korea National Health and Nutrition Examination Survey (KNHANES) provides a unique opportunity to address these gaps. KNHANES is a nationally representative survey employing a complex, multistage probability sampling design and standardized laboratory testing [22, 23]. The 2024 cycle is particularly valuable because it includes comprehensive biochemical measurements related to mineral metabolism, such as 25-hydroxyvitamin D [25(OH)D], Ca, Mg, P, and alkaline phosphatase (ALP), as well as bone mineral density assessment, enabling construction of a biologically informed reference population [22].

Therefore, the present study aimed to evaluate the reference interval of serum PTH in a nationally representative Korean population using KNHANES data, with explicit recognition of the assay-specific nature of PTH measurement. By applying a stepwise, biologically informed selection strategy and survey-weighted statistical methods, this study seeks to provide clinically relevant evidence for population- and assay-specific interpretation of PTH results in Korea.

## Methods

### Study design and data source

This cross-sectional study used data from the 2024 Korea National Health and Nutrition Examination Survey (KNHANES), a nationally representative survey of the non-institutionalized Korean population employing a complex, multistage, stratified probability sampling design [22, 23]. The survey comprises health interviews, physical examinations, and laboratory measurements conducted according to standardized protocols [22, 23]. For the present analysis, laboratory and health examination data from adult participants who underwent serum PTH measurement were extracted. All analyses were performed using de-identified public-use data provided by KNHANES.

### Study population and stepwise selection of the reference population

To establish a biologically appropriate reference population for PTH, a stepwise exclusion approach (Steps 0–11) was applied. The initial population (Step 0) included all participants with available serum PTH measurements. Subsequent steps progressively excluded participants with clinical conditions or laboratory findings known or suspected to influence PTH metabolism. At each step, only participants with non-missing serum PTH values were retained for further analyses. Although ALP is not mandatory for the assessment of PTH status, it may serve as an adjunctive marker of bone metabolic activity [24, 25].

The stepwise selection criteria were as follows:

- Step 1: Exclusion of participants with self-reported physician-diagnosed chronic diseases or abnormal health conditions.
- Step 2: Exclusion of participants with a history of dietary supplement use (≥ 2 weeks) within the preceding year.
- Step 3: Exclusion of pregnant participants.
- Step 4: Exclusion of participants with an irregular pulse identified during the physical examination.
- Step 5: Exclusion of participants with hypertension based on blood pressure measurements obtained during the survey examination.
- Step 6: Exclusion of participants classified as obese (body mass index ≥ 25 kg/m²).
- Step 7: Exclusion of participants with diabetes mellitus, defined by fasting plasma glucose ≥ 126 mg/dL, glycated hemoglobin (HbA1c) ≥ 6.5%, a physician diagnosis of diabetes mellitus, or current treatment with insulin or oral hypoglycemic agents.
- Step 8: Inclusion restricted to participants with an estimated glomerular filtration rate (eGFR) ≥ 60 mL/min/1.73 m².
- Step 9: Inclusion restricted to participants with serum 25(OH)D concentrations ≥ 20 ng/mL (50 nmol/L).
- Step 10: Inclusion restricted to participants with serum Ca, Mg, and P concentrations within predefined reference intervals provided by the assay manufacturers: 8.6–10.0 mg/dL for Ca, 1.6–2.6 mg/dL for Mg, and 2.5–4.5 mg/dL for P [22].
- Step 11: Inclusion restricted to participants with serum ALP concentrations within sex-specific reference intervals provided by the assay manufacturers: 40–129 U/L for men and 35–104 U/L for women [22].

There is no globally unified consensus regarding the optimal cutoff for total 25(OH)D concentration in relation to bone mineral health, and thresholds of 20 ng/mL (50 nmol/L) and 30 ng/mL (75 nmol/L) have been variably proposed [26–28]. In Korea, the Evidence-based Recommendations for Osteoporosis in Primary Care, published in 2024 by the Korean Academy of Medical Sciences in collaboration with the Korea Disease Control and Prevention Agency, recommend a target range of 30–50 ng/mL [26]. However, in the present study, applying a vitamin D deficiency cutoff of < 30 ng/mL (75 nmol/L) was expected to exclude more than three-quarters of the study population; therefore, a cutoff of < 20 ng/mL (50 nmol/L) was adopted [26–28].

### Laboratory measurements

Serum PTH and other biochemical parameters, including Ca, Mg, P, ALP, and 25(OH)D, were measured as part of the KNHANES health examination using standardized laboratory methods [22, 23]. Serum PTH concentrations were measured using the Elecsys PTH assay on the cobas e801 analyzer based on an electrochemiluminescence immunoassay principle (Roche Diagnostics, Mannheim, Germany) [22]. This assay is designed to measure intact PTH using a two-site sandwich immunoassay with monoclonal antibodies directed against defined epitopes of the PTH molecule [16–18, 22]. Serum Ca, Mg, P, and ALP concentrations were measured using the CA2, MG2, PHOS2, and ALP2 reagent kits on an automated cobas 8000 c702 analyzer (Roche Diagnostics, Mannheim, Germany) [20]. Serum 25(OH)D2 and 25(OH)D3 were simultaneously measured using a validated liquid chromatography–tandem mass spectrometry (LC-MS/MS) assay, and total 25(OH)D concentrations were calculated as the sum of these two components [20, 21]. When serum 25(OH)D2 concentrations were below the lower limit of quantification (0.3 ng/mL), values were imputed as 0.21 ng/mL (0.3/√2) in accordance with the KNHANES data analysis guidelines [20, 25]. All analytical procedures and quality control measures were performed centrally by the KNHANES reference laboratory in accordance with national standardized protocols [22]. The analytical quality of KNHANES laboratory data is monitored through formal quality control programs conducted by expert groups, including laboratory medicine specialists, and documented in official annual quality control reports [22, 23]. Therefore, the dataset used in this study was considered analytically consistent and suitable for indirect reference interval estimation. However, considering the known inter-assay variability in PTH measurement, the indirect reference interval estimated in this study is specific to the Elecsys PTH assay [7–18].

### Statistical analysis

Within each step-defined population, outliers were evaluated after restricting the analysis to participants with available serum PTH measurements [19, 20]. Outlier detection was performed using a robust statistical approach based on the median absolute deviation (MAD), and PTH values exceeding ± 3.5 MAD from the median were considered outliers [19–21, 28]. Outlier removal was applied exclusively to PTH values (PTH-only rule) to avoid unnecessary exclusion of participants due to unrelated biochemical variables. This study was conducted in accordance with the official Guidebook for Data Users of the KNHANES [22]. Because KNHANES uses a multistage, stratified probability-sampling design, all weighted analyses incorporated the primary sampling unit (psu), stratification variable (kstrata), and examination weights (wt_itvex) to generate nationally representative estimates [22]. Primary sampling units (psu), stratification variables (kstrata), and examination weights (wt_itvex) were incorporated to account for unequal selection probabilities and non-response in the complex survey design [22]. Within each step-defined and outlier-cleaned population, PTH reference intervals were estimated as the 2.5th to 97.5th percentiles [17, 19]. Percentiles were calculated using survey-weighted quantile estimation to reflect the nationally representative distribution of serum PTH concentrations. For subgroups with fewer than 120 observations, a robust method aligned with CLSI EP28-A3c was applied to the unweighted PTH values after step-specific restriction and outlier exclusion, and 90% confidence intervals for the lower and upper reference limits were estimated by bootstrap resampling [19]. For comparison purposes, unweighted percentile estimates were also calculated. Assay-specific reference intervals for PTH were reviewed in conjunction with information reported in the literature. Descriptive statistics, including the median and percentile distributions of PTH, were calculated for each step. Statistical analyses were performed using R version 4.3.2 (R Foundation for Statistical Computing, Vienna, Austria) and the survey package (version 4.4-2), with svydesign used for survey design specification and svyquantile for weighted percentile estimation.

## Results

### Study population and stepwise selection

Of the 6,997 participants in the KNHANES 2024 dataset, serum PTH measurements were available for 4,502 adults aged ≥ 40 years. As progressively restrictive selection criteria were applied from Steps 1 through 11, the number of eligible participants decreased sequentially (Table 1). The most substantial reductions in sample size occurred after exclusions based on disease history (Step 1), dietary supplement use (Step 2), obesity (Step 6), and vitamin D deficiency (Step 9). After excluding participants with missing PTH values and applying step-specific eligibility criteria, Step 11 yielded the smallest yet most stringently defined reference population, representing individuals without apparent conditions affecting calcium–phosphate metabolism.

**Table 1:** Number of subjects according to stepwise selection criteria

Step	Rule	*N* of residual subjects	*N* of removed subjects	*N* of subjects with PTH measurement	*N* of men	*N* of women
0	Start (all survey subjects)	6,997	0	4,502	1,908	2,594
1	Exclusion of participants with self-reported physician-diagnosed chronic diseases or abnormal health conditions	2,170	4,827	861	379	482
2	Exclusion of participants with a history of dietary supplement use (≥ 2 weeks) within the preceding year	868	1,302	288	156	132
3	Exclusion of pregnant participants	867	1	288	156	132
4	Exclusion of participants with an irregular pulse identified during the physical examination	861	6	283	153	130
5	Exclusion of participants with hypertension based on blood pressure measurements obtained during the survey examination	795	66	235	116	119
6	Exclusion of participants classified as obese (body mass index ≥ 25 kg/m²)	635	160	165	71	94
7	Exclusion of participants with diabetes mellitus, defined by fasting plasma glucose ≥ 126 mg/dL, glycated hemoglobin ≥ 6.5%, a physician diagnosis of diabetes mellitus, or current treatment with insulin or oral hypoglycemic agents	632	3	162	69	93
8	Inclusion restricted to participants with an estimated glomerular filtration rate ≥ 60 mL/min/1.73 m²	489	143	159	66	93
9	Inclusion restricted to participants with serum 25-hydroxyvitamin D concentrations ≥ 20 ng/mL	84	405	84	37	47
10	Inclusion restricted to participants with serum calcium, magnesium, and phosphorus concentrations within predefined reference intervals provided by the assay manufacturers	83	1	83	37	46
11	Inclusion restricted to participants with serum alkaline phosphatase concentrations within sex-specific reference intervals provided by the assay manufacturers	82	1	82	36	46

Abbreviations: N, number; PTH, parathyroid hormone

### Outlier detection and survey-weighted reference interval estimation

Within each step-defined population, outlier assessment was conducted among participants with available serum PTH measurements. Using a robust median absolute deviation–based approach, a small proportion of extreme PTH values were identified and excluded. The number of excluded outliers was limited across all steps, and the overall distribution of PTH concentrations remained stable following outlier removal. The numbers of participants before and after outlier exclusion at each step are summarized in Supplementary Table S1 and Table 2.

**Table 2:** Age (years) and parathyroid hormone levels (ng/mL) of subjects according to stepwise selection criteria

Step	Rule (abbr.)	Characteristics	Unweighted, before outlier exclusion							Weighted, after outlier exclusion						
			N	Mean	SD	Med	IQR	2.5th-97.5th	Min-Max	N	Mean	SD	Med	IQR	2.5th-97.5th	Min-Max
0	Start	Age	4502	61.1	11.8	62.0	52.0–71.0	41.0–80.0	40.0–80.0	4408	58.7	11.6	58.0	49.0–67.0	41.0–80.0	40.0–80.0
		PTH	4502	39.2	16.1	36.4	29.4–45.5	18.7–74.6	5.4–352.0	4408	37.8	11.9	36.0	29.3–44.9	19.1–66.1	5.4–77.2
1	Exclusion of chronic diseases	Age	861	54.5	10.9	53.0	45.0–62.0	40.0–80.0	40.0–80.0	845	52.9	10.1	51.0	44.0–59.0	40.0–80.0	40.0–80.0
		PTH	861	39.3	14.1	36.9	30.0–46.1	20.4–72.3	12.8–169.0	845	38.3	11.4	36.5	30.0–45.3	20.5–63.5	12.8–76.6
2	Exclusion of dietary supplement use	Age	288	55.2	12.1	53.0	45.0–64.0	40.0–80.0	40.0–80.0	282	53.1	11.0	51.0	44.0–58.0	40.0–80.0	40.0–80.0
		PTH	288	41.7	16.8	38.7	32.0–48.5	21.0–78.1	17.4–169.0	282	40.8	12.5	38.6	32.5–48.9	21.0–74.0	17.4–78.7
3	Exclusion of pregnancy	Age	288	55.2	12.1	53.0	45.0–64.0	40.0–80.0	40.0–80.0	282	53.1	11.0	51.0	44.0–58.0	40.0–80.0	40.0–80.0
		PTH	288	41.7	16.8	38.7	32.0–48.5	21.0–78.1	17.4–169.0	282	40.8	12.5	38.6	32.5–48.9	21.0–74.0	17.4–78.7
4	Exclusion of arrythmia	Age	283	55.0	12.0	53.0	45.0–63.0	40.0–80.0	40.0–80.0	277	52.9	10.9	51.0	44.0–58.0	40.0–80.0	40.0–80.0
		PTH	283	41.7	16.9	38.7	32.1–48.3	21.0–78.2	17.4–169.0	277	40.8	12.5	38.7	32.5–48.9	20.5–74.0	17.4–78.7
5	Exclusion of hypertension	Age	235	54.4	11.7	52.0	44.0–62.5	40.0–80.0	40.0–80.0	232	52.5	10.6	50.0	44.0–58.0	40.0–80.0	40.0–80.0
		PTH	235	41.1	15.6	38.3	31.6–47.6	21.0–77.3	18.8–169.0	232	40.9	13.1	38.6	31.7–48.6	21.0–77.1	18.8–80.8
6	Exclusion of obesity	Age	165	54.9	11.8	53.0	45.0–63.0	40.0–80.0	40.0–80.0	160	53.1	10.8	51.0	44.0–59.0	40.0–80.0	40.0–80.0
		PTH	165	40.7	16.6	37.1	31.3–46.5	21.1–78.2	18.8–169.0	160	39.0	11.8	36.9	30.9–45.6	20.5–67.7	18.8–77.1
7	Exclusion of diabetes mellitus	Age	162	54.8	11.9	52.5	45.0–63.0	40.0–80.0	40.0–80.0	157	53.0	10.9	51.0	44.0–59.0	40.0–80.0	40.0–80.0
		PTH	162	40.9	16.7	37.1	31.4–46.6	21.9–78.3	18.8–169.0	157	39.2	11.8	37.0	31.3–46.5	20.5–67.7	18.8–77.1
8	Inclusion of eGFR ≥ 60 mL/min/1.73 m²	Age	159	54.7	11.8	52.0	45.0–63.0	40.0–80.0	40.0–80.0	155	52.9	10.8	51.0	44.0–59.0	40.0–80.0	40.0–80.0
		PTH	159	40.1	13.4	37.1	31.4–46.6	21.9–77.2	18.8–90.6	155	39.2	11.9	37.1	31.0–46.5	20.5–67.7	18.8–77.1
9	Inclusion of 25(OH)D ≥ 20 ng/mL	Age	84	56.3	11.8	55.0	45.8–65.0	40.0–80.0	40.0–80.0	82	53.8	10.9	52.0	44.0–60.0	40.0–80.0	40.0–80.0
		PTH	84	36.8	11.5	33.5	27.9–44.0	21.9–64.3	18.8–75.0	82	36.8	10.8	34.3	27.7–45.1	20.5–62.4	18.8–64.5
10	Inclusion of normal Ca, Mg, and P levels	Age	83	56.3	11.9	55.0	45.5–65.0	40.0–80.0	40.0–80.0	81	53.7	11.0	52.0	44.0–61.0	40.0–80.0	40.0–80.0
		PTH	83	36.8	11.5	33.4	27.9–44.4	21.9–64.4	18.8–75.0	81	36.8	10.8	34.3	27.7–45.3	20.5–62.4	18.8–64.5
11	Inclusion of normal ALP levels	Age	82	56.4	12.0	55.0	45.3–65.0	40.0–80.0	40.0–80.0	80	53.8	11.1	52.0	44.0–61.0	40.0–80.0	40.0–80.0
		PTH	82	36.9	11.6	33.5	27.9–44.7	21.9–64.5	18.8–75.0	80	37.0	10.9	35.1	27.7–45.3	20.5–62.4	18.8–64.5

Abbreviations: abbr., abbreviated; ALP, alkaline phosphatase; Ca, calcium; eGFR, estimated glomerular filtration rate; IQR, interquartile range; Max, maximum; Med, median; Mg, magnesium; Min, minimum; N, number; P, phosphate; PTH, parathyroid hormone; SD, standard deviation

Survey-weighted percentile estimation incorporating sampling weights, stratification variables, and primary sampling units was performed for each step-defined population after outlier removal. The number of survey-design–valid participants contributing to weighted estimation decreased progressively with increasing step number. Survey-weighted reference intervals (2.5th–97.5th percentiles) were successfully estimated for early and intermediate steps. However, for Steps 9–11, the number of survey-design–valid participants frequently fell below the predefined threshold of 120, precluding reliable estimation of survey-weighted reference intervals in some steps.

**Fig. 1:**
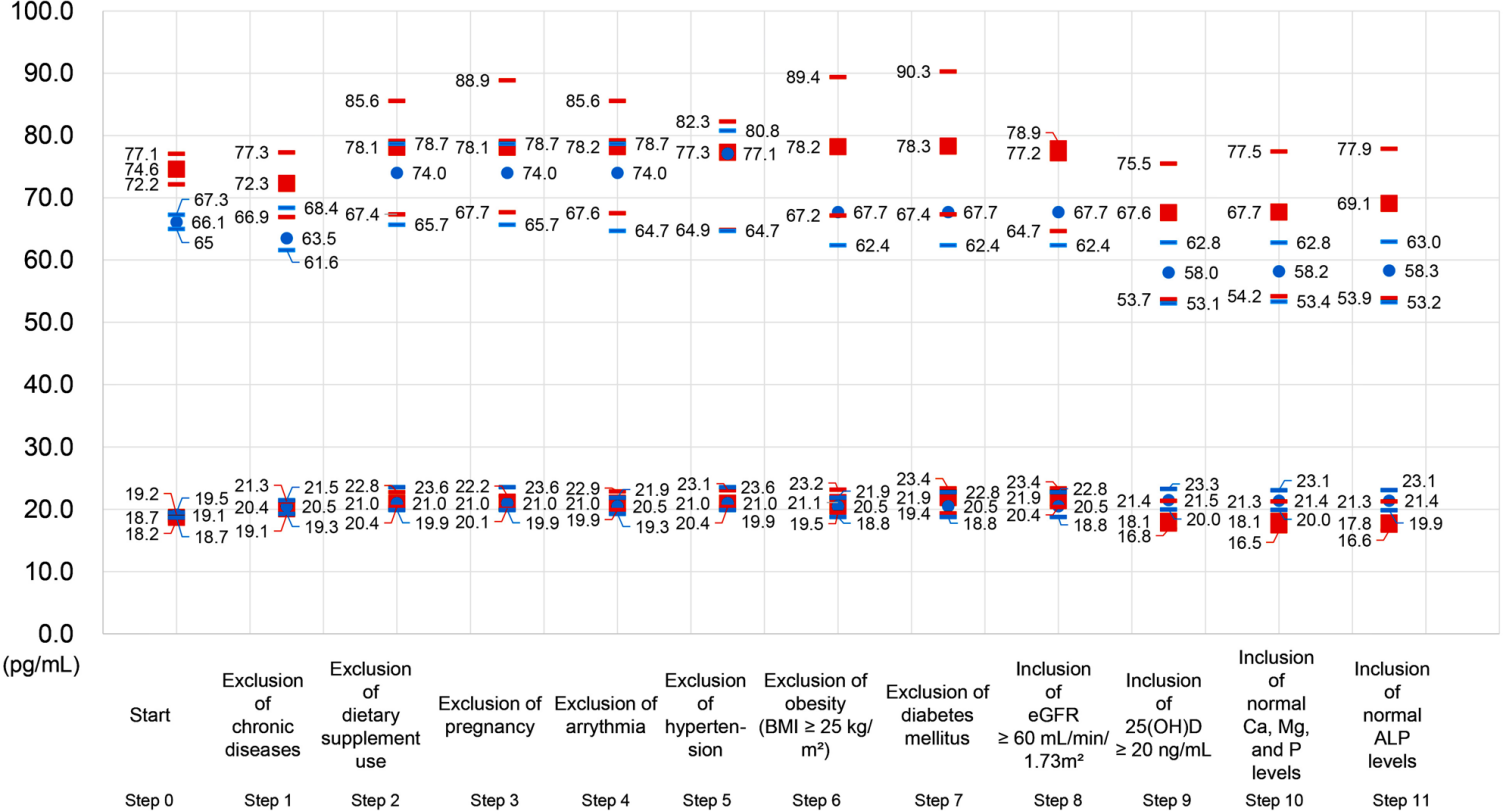
The 2.5th to 97.5th percentile levels of parathyroid hormone according to the stepwise selection criteria, before and after outlier removal and application of survey weights. The orange circles represent unweighted levels before outlier removal, and the blue circles represent weighted levels after outlier removal. The 90% confidence intervals are shown as solid lines in the same color as the corresponding markers. The unweighted level before outlier exclusion is displayed as a number to the left of each marker, and the weighted level after outlier exclusion is displayed as a number to the right of each marker. The missing upper confidence bound in Steps 6–8 likely reflects instability of survey-weighted quantile interval estimation in the extreme upper tail, where only a few highly weighted observations determined the 97.5th percentile. Abbreviations: 25(OH)D, 25-hydroxyvitamin D; ALP, alkaline phosphatase; BMI, body mass index; Ca, calcium; eGFR, estimated glomerular filtration rate; P, phosphate; PTH, parathyroid hormone

Unweighted percentile estimates were calculated in parallel for visual comparison (Fig. 1). Across most steps, unweighted and survey-weighted 2.5th value of PTH concentrations were similar. However, the survey-weighted 97.5th percentile of PTH exhibited greater variability than the unweighted estimate across steps. After excluding participants using dietary supplements and those with obesity (BMI ≥ 25 kg/m²), the effective sample size declined markedly, indicating increased heterogeneity in sampling weights within the restricted subpopulation. As progressively stricter biological criteria were applied, the width of the PTH reference interval narrowed.

### Final reference population

Step 11, which incorporated renal function, vitamin D sufficiency, mineral balance, age restriction, and sex-specific ALP criteria, represented the most physiologically well-defined reference population. However, due to the reduced sample size at this step, reference interval estimates derived from Step 11 did not meet the recommended minimum sample size threshold (< 120). Although PTH levels varied according to vitamin D status, the number of subjects was insufficient (< 120) to adequately establish vitamin D–specific PTH reference intervals.

Among 155 participants with no history of disease, no dietary supplement use, non-pregnant status, and an estimated glomerular filtration rate ≥ 60 mL/min/1.73 m², the unweighted and survey-weighted 2.5th–97.5th reference intervals for PTH were 21.8–65.0 pg/mL (2.3–6.9 pmol/L) and 20.5–67.7 pg/mL (2.2–7.2 pmol/L), respectively, when vitamin D deficiency status and serum calcium, magnesium, and phosphate levels were not considered. These results were reviewed in conjunction with previously reported PTH reference intervals obtained using assays from the same manufacturer and are summarized in Table 3.

**Table 3:** Parathyroid hormone levels (ng/mL) measured using the Elecsys PTH assay (Roche Diagnostics, Manheim, Germany)

Reference	Category	*N* of subjects	PTH 2.5th	PTH 97.5th
Manufacturer’s information^†^	Previous information from the instructions for use	N/A	16.0	65.0
	Subjects with normal Ca, P, and creatinine levels, regardless of 25(OH)D status	489	17.3	74.1
	Subjects with normal Ca, P, and creatinine levels and 25(OH)D ≥ 30 ng/mL	141	17.9	58.6
	Subjects with normal Ca, P, and creatinine levels and 25(OH)D > 20 to < 30 ng/mL	165	17.0	60.4
	Subjects with normal Ca, P, and creatinine levels and 25(OH)D ≤ 20 ng/mL	183	19.5	86.4
Gong and Wang et al.^†^ [15]	Subjects with Ca 8.4–10.4 mg/dL, P 2.5–4.5 mg/dL, eGFR ≥ 60 mL/min/1.73m^2^, and 25(OH)D ≥ 20 ng/mL	25,139	22.3	73.2
	Subjects with Ca 8.4–10.4 mg/dL, P 2.5–4.5 mg/dL, eGFR ≥ 60 mL/min/1.73m^2^, and 25(OH)D ≥ 30 ng/mL	4,465	21.5	68.5
Hataysal et al.^‡^ [16]	Subjects with Ca 8.4–10.4 mg/dL, P 2.5–4.5 mg/dL, and eGFR ≥ 60 mL/min/1.73m^2^, regardless of 25(OH)D status	22,662	15.3	76.8
	Subjects with Ca 8.4–10.4 mg/dL, P 2.5–4.5 mg/dL, eGFR ≥ 60 mL/min/1.73m^2^, and 25(OH)D > 20 to < 30 ng/mL	11,356	15.6	78.1
Choi et al.^§^ [29]	Subjects with Ca 8.6–10.0 mg/dL, P 2.5–4.5 mg/dL, and eGFR ≥ 60 mL/min/1.73m^2^, regardless of 25(OH)D status	449	17.3	53.8
	Subjects with Ca 8.6–10.0 mg/dL, P 2.5–4.5 mg/dL, eGFR ≥ 60 mL/min/1.73m^2^, and 25(OH)D ≥ 30 ng/mL	254	16.6	50.1
	Subjects with Ca 8.6–10.0 mg/dL, P 2.5–4.5 mg/dL, eGFR ≥ 60 mL/min/1.73m^2^, and 25(OH)D ≥ 20 ng/mL	356	16.8	52.1
This study^||^	Subjects with eGFR ≥ 60 mL/min/1.73m^2^, regardless of 25(OH)D status (survey–weighted)	155	20.5	67.7
	Subjects with eGFR ≥ 60 mL/min/1.73m^2^ and 25(OH)D levels ≥ 20 ng/mL (survey–weighted)	82^¶^	21.5	58.0
	Subjects with Ca 8.6–10.0 mg/dL, P 2.5–4.5 mg/dL, eGFR ≥ 60 mL/min/1.73m^2^, and 25(OH)D ≥ 20 ng/mL(survey–weighted)	81^¶^	21.4	58.2

Abbreviations: 25(OH)D, 25-hydroxyvitamin D; Ca, calcium; eGFR, estimated glomerular filtration rate; N/A, not available; P, phosphate; PTH, parathyroid hormone

Serum 25-hydroxyvitamin D [25(OH)D] was measured using the ^†^Cobas e601 assay (Roche Diagnostics, Mannheim, Germany) [15], the ^‡^Cobas 8000 system (Roche Diagnostics, Mannheim, Germany) [16], the ^§^ARCHITECT i2000SR assay (Abbott Diagnostics, Abbott Park, IL, USA) [29], and the ^||^LC-MS/MS method [25]. ^¶^For subgroups with fewer than 120 observations, a robust method aligned with CLSI EP28-A3c was applied to the unweighted PTH values after step-specific restriction and outlier exclusion, and 90% confidence intervals for the lower and upper reference limits were estimated by bootstrap resampling [17]. The manufacturer’s information sheet described calcium, phosphate, and creatinine as normal, but did not provide detailed numerical values. When serum calcium and phosphate concentrations were reported in mmol/L, conversion factors of 4.01 (mmol/L × 4.01 = mg/dL) for calcium and 3.10 (mmol/L × 3.10 = mg/dL) for phosphate were applied

## Discussion

In this study, we evaluated the reference interval of serum PTH in a nationally representative Korean population using data from the KNHANES. To our knowledge, few studies have systematically evaluated PTH reference intervals in the Korean population, particularly using a stepwise, biologically informed approach combined with survey-weighted analysis [29]. Given the well-recognized analytical and biological variability of PTH assays, the lack of population-specific reference interval data represents an important gap in clinical laboratory practice.

The present analysis highlights the methodological challenges inherent in establishing PTH reference intervals using population-based datasets [19–21]. Although the KNHANES 2024 survey provides a uniquely valuable resource by including both biochemical measurements and bone mineral density assessments, its applicability for reference interval estimation was constrained by several factors. In particular, the high prevalence of dietary supplement use in the Korean population substantially reduced the size of an appropriate reference population after biologically relevant exclusions were applied [15, 19, 30, 31]. The reduction in effective sample size after excluding participants with obesity suggests increased heterogeneity in sampling weights [32–34]. Because upper tail quantiles in complex survey data are sensitive to weight variability and domain restriction, differences between weighted and unweighted reference limits may reflect sampling structure rather than biological variation [32]. Accordingly, effective sample size and weight distribution should be considered when estimating survey-based reference intervals [22, 23, 32]. When supplement use and related biochemical criteria were considered simultaneously, the number of eligible participants decreased to levels below commonly recommended thresholds for reference interval estimation [15, 19]. The influence of reduced sample size should be considered when interpreting these changes [32]. Overall, these findings align with current physiological understanding and reinforce recommendations that PTH reference intervals should not be established in unselected populations [15–18, 29, 35]. In the present study, despite the substantial reduction in sample size across steps, the derived reference intervals changed only modestly, suggesting that the cumulative impact of the exclusions was limited. Reporting confidence intervals for the lower and upper reference limits may further clarify the precision of these estimates and help identify which exclusion criteria had the greatest influence on the observed changes in the reference interval. However, because the final study population became progressively more restricted and likely healthier than the general population, potential selection bias and limited generalizability should be considered when interpreting the final reference interval.

An additional practical challenge in establishing PTH reference intervals in Korea relates to healthcare reimbursement policies and test utilization patterns [36]. In the Korean healthcare system, serum PTH measurement is subject to specific reimbursement criteria and is not routinely included in general health screening for apparently healthy individuals [37]. Consequently, PTH testing in clinical practice is typically performed only when clinically indicated, rather than as part of broad population-based health examinations [36]. Moreover, the analytical cost of PTH testing is substantially higher than that of routine chemistry analytes, limiting its availability in large-scale screening settings [19]. For these reasons, studies evaluating PTH reference intervals using residual clinical specimens are rare, as such samples are generally derived from patients undergoing targeted diagnostic evaluation rather than from healthy populations [19]. Collectively, these factors constrain the feasibility of traditional direct reference interval studies based on residual specimens [19–21]. As a result, PTH represents an analyte for which indirect methods of reference interval estimation using large clinical datasets are particularly challenging [20, 21]. The selective nature of test ordering, combined with reimbursement restrictions and higher analytical costs, introduces substantial selection bias into routine laboratory databases, making it difficult to identify an appropriate reference population using indirect approaches [19–21]. In this context, nationally representative survey data with standardized laboratory measurements, such as KNHANES, provide a uniquely valuable alternative for reference interval evaluation, despite inherent limitations related to sample size after biologically relevant exclusions.

A key limitation of this study relates to age coverage. In the 2024 KNHANES cycle, bone-related examinations, including bone mineral density measurements, were restricted to participants aged 40 years and older. Consequently, the reference interval estimates derived in this study primarily reflect middle-aged and older adults and cannot be directly extrapolated to younger populations. However, previous studies have reported no substantial differences in PTH reference intervals between younger and middle-aged adults [17, 18, 29]. This suggests that the reference interval derived in the present study may still be broadly applicable to the adult population. Furthermore, the close agreement between our estimated reference interval and the manufacturer’s recommended interval further supports the potential validity of our findings. A nationally coordinated platform, such as the National Health Screening Program, could provide a practical framework for establishing reference intervals in younger populations through the integration of direct and indirect methods, assay-specific verification, and age-continuous modeling [19, 29]. Future studies in younger populations could benefit from approaches used in international pediatric reference interval initiatives, including multicenter recruitment of healthy children, age- and sex-specific partitioning, assay-specific validation, and harmonization across analytical platforms [38].

Despite these limitations, this study provides important insights into the interpretation of PTH concentrations in the context of bone health. The integration of biochemical markers with bone-related outcomes in a nationally representative sample suggests that future analyses could extend beyond reference interval estimation to provide clinically relevant information on the relationships among PTH, mineral metabolism, and skeletal health across different age groups [15]. Such analyses may have implications not only for laboratory reference interval setting but also for risk stratification and public health strategies related to bone health in the Korean population.

## Conclusions

In conclusion, this study demonstrates that establishing reference intervals for PTH requires a biologically informed and methodologically rigorous approach that accounts for renal function, vitamin D status, and mineral metabolism. Although KNHANES 2024 provides a robust foundation for such analyses, limitations related to dietary supplement use and age coverage underscore the need for larger and more diverse reference populations. Future population-based studies incorporating a broader age range and comprehensive biochemical profiling will be essential to refine PTH reference intervals and to clarify their broader implications for bone health in Korea.

## Electronic Supplementary Material

Below is the link to the electronic supplementary material.

Supplementary Material 1

## Data Availability

The datasets generated and analyzed during the current study are available from the public database of KNHANES https://knhanes.kdca.go.kr/knhanes/eng/main.do.

## Abbreviations

25(OH)D: 25-hydroxyvitamin D
ALP: Alkaline phosphatase
Ca: Calcium
eGFR: Estimated glomerular filtration rate
HbA1c: Glycated hemoglobin
KNHANES: Korea National Health and Nutrition Examination Survey
MAD: Median absolute deviation
Mg: Magnesium
P: Phosphate
PTH: Parathyroid hormone
